# Perspectives on Using Online Platforms for Promoting Running and Walking Activities

**DOI:** 10.3389/fpubh.2020.00150

**Published:** 2020-04-28

**Authors:** Apichai Wattanapisit, Waluka Amaek, Naparat Sukkriang, Sanhapan Wattanapisit, Sunton Wongsiri

**Affiliations:** ^1^School of Medicine, Walailak University, Nakhon Si Thammarat, Thailand; ^2^Department of Clinical Medicine, Walailak University Hospital, Nakhon Si Thammarat, Thailand; ^3^Walailak University Running for Health Club, Nakhon Si Thammarat, Thailand; ^4^School of Architecture and Design, Walailak University, Nakhon Si Thammarat, Thailand; ^5^Department of Family Medicine, Thasala Hospital, Nakhon Si Thammarat, Thailand; ^6^Department of Orthopedic Surgery and Physical Medicine, Faculty of Medicine, Prince of Songkla University, Songkhla, Thailand

**Keywords:** exercise, health promotion, physical activity, technology, virtual run

## Introduction

Physical inactivity is a global health burden (1, 2). The World Health Organization targets to reduce the prevalence of physical inactivity by 15% by 2030 (3). Several tools and methods, such as virtual peer groups; virtual environments; virtual and augmented reality technologies; exergames or gamification, have been used to promote physical activity (PA) in the digital age (4–7). A systematic review reveals that using technology-based interventions is more effective than non-technology interventions in promoting PA (8). The development of technology is changing human interactions with physical and virtual environments (9). Traditionally, people perform PA in the real environments. However, technologies can facilitate people to perform PA in different environments (i.e., real, mixed reality, or virtual environments) (9). Moreover, virtual reality (VR) technologies or virtual platforms are used to promote PA (7, 10). According to the growth of VR and technology, the word “virtual” has been more popular.

A virtual run or virtual race is an example of the use of the word “virtual.” The definition of virtual run is uncertain and is not well-clarified in scientific literature. The Google search using the term “virtual run” or “virtual race” found a variety of search results, for example, running event websites, advertisements of virtual run programmes, video clips about virtual runs, news about virtual runs. An online newspaper reported an increase in popularity of virtual runs in the past few years (11). According to the search, a virtual run is a running activity using an online platform to record activities. In other words, a virtual run needs a real running activity and an online recording platform.

A virtual run programme is not limited to a specific software. Generally, a virtual run programme consists of three main steps: (i) select a running activity or a race; (ii) run/walk and record the activities; and (iii) submit the results to the organiser via the online platform (12). Nevertheless, the details and methods of each virtual run programme are different. A virtual run organiser creates instructions and an online platform (13, 14). A virtual run programme allows the participant to run or walk at any location (e.g., on a road, on a track, on a treadmill) and at any time. The activity can be a single run (e.g., 5, 10 km, half marathon, marathon) or a multi-session run (e.g., 100 km in a month). A participant needs a device to record the running or walking activities and connect to the organised platform.

Recording and submitting methods vary among virtual run programmes (13, 14). For example, participants can record their running history on a mobile or wearable device, as well as by taking a photo of the results on a treadmill screen. To submit the running results, some organisers provide a web-based platform where participants can submit their results. Some virtual run activities offer a real-time platform where participants can accumulate the total running distance or time on a mobile application. Moreover, some platforms allow participants to see peers' results (15, 16). The final goal of a virtual run activity is to achieve a distance-based (e.g., 100 km in a month) or time-based target (e.g., 8 h in a month) (13). Some virtual run organisers offer rewards, such as medals, tokens, or running jerseys, for those achievements (17). Some activities are free. Others are paid for by charities or are commercial (18).

## Perspectives on Virtual Run Programmes

A virtual run programme seems to be a novel intervention, in terms of its processes. However, it has some similar characteristics of other interventions or methods. For example, a virtual run encourages participants to run with a challenge, and uses an online platform to record the running activities. The evidence supports the positive effects of using a combination of behavioural challenges and PA trackers on health outcomes (19). To the best of our knowledge, research on the virtual run programmes is sparse. This article aims to debate the rationale and potential of virtual run programmes for promoting PA.

### Rationale 1: Promoting Physical Activity Through Running or Walking Activities

Virtual run programmes instruct participants to run, jog, or walk. Participants can perform real activities in a real or virtual environment or in a combination of real and virtual environments. For example, a participant can walk on a treadmill while watching a video clip on a mobile device that shows a first-person view of an attractive setting for 1 h.

### Rationale 2: Setting a Clear Goal

Virtual run programmes set goals in the activities. The goals can be distance-based or time-based targets (13). The target of a virtual run programme is clear and knowable prior to beginning the programme. Many virtual run programmes are arranged for non-profit purposes, while others are commercial programmes. This can be a goal of the virtual run participants, for example, running for charity (18). Some virtual run programmes offer rewards or incentives for activity completion (17). Goal setting is one of the effective behaviour change techniques (20).

### Rationale 3: Using an Online Platform

Virtual run programmes are operated by using online platforms (i.e., websites, mobile applications, social media). The online platforms are tools for recording and monitoring participants' activities. Self-monitoring of activities is part of self-regulation, which can promote behaviour change (21, 22). Participants can record their activities by using Global Positioning System (GPS) tracking via mobile or wearable devices, pedometers, or accelerometers. Otherwise, participants can manually record their activity distance and/or activity time on the platforms. Some virtual run programmes allow participants to monitor their own activities as well as those of their peers.

## Discussion

Future research should focus on the impacts of virtual run programmes on health outcomes, implementation, and pragmatic examinations of virtual runs. According to the limited evidence, the rationale and potential of using virtual run programmes for promoting PA were debated. Virtual run programmes may offer some potential advantages. In addition, challenges and recommendations were addressed.

### Advantages

Virtual run programmes can promote PA through running or walking activities. Running and walking are uncomplicated and convenient activities (23–25). Running and walking can decrease several health risks, such as waist circumference, body weight, risks of non-communicable diseases (NCDs), and premature deaths (25–27). Moreover, running, and walking can benefit mental health (28, 29). Most importantly, virtual run programmes can offer instructed and flexible activities. For example, participants can walk and run in their preferred environments at convenient times.

Participants can select a suitable virtual run programme based on their goals. A goal-setting process is useful to help people change their behaviours or lifestyles to become healthier (30). A virtual run programme can play the role of coach, and can advocate a SMART goal (Specific, Measurable, Achievable, Results-focused, Timely) (31). A SMART goal can be an effective strategy to improve physical fitness (32). A virtual run programme can instruct running or walking (specific), record distance or time (measurable), offer a variety of programmes for different individuals (achievable), set a realistic result (result-focused), and set a realistic timeframe (timely). Moreover, an incentive-based intervention can be a promising approach for promoting PA (33).

Using online platforms can reach a large number of people (34, 35). Moreover, using online-delivered interventions, including websites and online social networks, can lead to positive behavioural outcomes (36, 37). This approach has the potential for promoting PA in the population. Virtual run programmes propose objectively measured PA. In a real-world setting, virtual run participants can use personal mobile or wearable devices to objectively record their PA (38–41). The real-time or final results of each participant are monitored. Some virtual run programmes create social network platforms, which allow participants in the networks to see peers' results. This can be beneficial in eliciting the Köhler effect, which is a reaction within a group whereby a weaker member tends to be more motivated by the proximity of a more capable partner. Studies show that the Köhler effect, in online exercise teams or exercise with virtually present and superior partners, can improve time spent PA and motivation (42, 43). The Köhler effect has been found in PA-promoting approaches in both real-world settings and digital platforms (42, 44, 45).

### Challenges

Running has a higher risk of injuries compared with walking (46). Competitive runs cause more injuries than recreational runs (47). Participating in virtual run programmes without preparation and concerns about health conditions may lead to adverse events. However, appropriate training can lower the risk of injuries (48, 49). Specifically, virtual run programmes, which mandate participants to carry mobile devices for recording and monitoring the activity, may cause a distraction during activities (50, 51).

Although a virtual run intervention can set a SMART goal, this can be challenging. A general message (e.g., 100 km in 3 months) may be a difficult task for some participants. It requires detailed information to guide participants to identify the goal, including both outcomes and behaviours (52, 53). Another issue is some virtual run programmes require payment for participation (54).

Although a virtual run programme can reach a wide range of populations, implementing and participating in a virtual run programme requires specific technology. A virtual run programme requires a device to record the activities. The devices can be GPS-based, internet-based, wireless, or wired. The connection to the platforms can be wireless (e.g., Bluetooth), or wired (i.e., using physical cables to transfer data). Most importantly, the process requires an internet connection to automatically or manually upload the activity records to the assigned platforms.

### Recommendations

Virtual run organisers should provide participants with the necessary information, including the appropriate levels of PA for individuals, how to prevent injuries, and specific precautions to take. Moreover, a self-health screening should be taken prior to a virtual run programme. Participants should be aware of their health conditions and readiness to participate in any virtual run programme. In addition, participants should focus on safety issues while participating in a virtual run programme.

Virtual run organisers should offer clear information. For example, the organisers provide a running distance and time window as well as rewards. This approach can help participants identify their appropriate goals. Virtual run organisers are expected to balance the incentive (a motivator) and the financial cost (a barrier) to support participants' decision-making. Participants should select a suitable and affordable programme to achieve their SMART goals.

Virtual run organisers should provide user-friendly platforms (e.g., web-based platforms, mobile applications) that support a variety of devices: for example, a platform that is able to connect to any brand of mobile or wearable devices through real-time online or offline bases. Participants who participate in the running activities while offline can subsequently transfer their records to the platform. Organisers can take the opportunity to use the technology to objectively measure PA instead of using subjective measurements. The social networks within the virtual run programmes should be organised to achieve the Köhler effect or intergroup motivation. Lastly, participants should understand the system of a virtual run programme and choose one that is suitable, which accommodates their personal devices.

## Author Contributions

AW and WA initiated the debate topic. AW, WA, NS, SWa, and SWo participated in discussions. AW wrote the first draft of the manuscript. All authors read and approved the final manuscript.

## Conflict of Interest

The authors declare that the research was conducted in the absence of any commercial or financial relationships that could be construed as a potential conflict of interest.
